# Seasonal monitoring of deep-sea megabenthos in Barkley Canyon cold seep by internet operated vehicle (IOV)

**DOI:** 10.1371/journal.pone.0176917

**Published:** 2017-05-30

**Authors:** Carolina Doya, Damianos Chatzievangelou, Nixon Bahamon, Autun Purser, Fabio C. De Leo, S. Kim Juniper, Laurenz Thomsen, Jacopo Aguzzi

**Affiliations:** 1 Instituto de Ciencias del Mar (ICM-CSIC), Barcelona, Spain; 2 Jacobs University Bremen, Bremen, Germany; 3 Centro de Estudios Avanzados de Blanes (CEAB-CSIC), Blanes, Spain; 4 Deep Sea Ecology and Technology group, Alfred-Wegener-Institute (AWI), Bremerhaven, Germany; 5 Ocean Networks Canada, University of Victoria, Victoria, BC, Canada; 6 Department of Biology, University of Victoria, Victoria, BC, Canada; UPMC, FRANCE

## Abstract

Knowledge of the processes shaping deep-sea benthic communities at seasonal scales in cold-seep environments is incomplete. Cold seeps within highly dynamic regions, such as submarine canyons, where variable current regimes may occur, are particularly understudied. Novel Internet Operated Vehicles (IOVs), such as tracked crawlers, provide new techniques for investigating these ecosystems over prolonged periods. In this study a benthic crawler connected to the NEPTUNE cabled infrastructure operated by Ocean Networks Canada was used to monitor community changes across 60 m^2^ of a cold-seep area of the Barkley Canyon, North East Pacific, at ~890 m depth within an Oxygen Minimum Zone (OMZ). Short video-transects were run at 4-h intervals during the first week of successive calendar months, over a 14 month period (February 14^th^ 2013 to April 14^th^ 2014). Within each recorded transect video megafauna abundances were computed and changes in environmental conditions concurrently measured. The responses of fauna to environmental conditions as a proxy of seasonality were assessed through analysis of abundances in a total of 438 video-transects (over 92 h of total footage). 7698 fauna individuals from 6 phyla (Cnidaria, Ctenophora, Arthropoda, Echinodermata, Mollusca, and Chordata) were logged and patterns in abundances of the 7 most abundant taxa (i.e. rockfish Sebastidae, sablefish *Anoplopoma fimbria*, hagfish *Eptatretus stoutii*, buccinids (Buccinoidea), undefined small crabs, ctenophores *Bolinopsis infundibulum*, and Scyphomedusa *Poralia rufescens*) were identified. Patterns in the reproductive behaviour of the grooved tanner crab (*Chionnecetes tanneri*) were also indicated. Temporal variations in biodiversity and abundance in megabenthic fauna was significantly influenced by variabilities in flow velocity flow direction (up or down canyon), dissolved oxygen concentration and month of study. Also reported here for the first time are transient mass aggregations of grooved tanner crabs through these depths of the canyon system, in early spring and likely linked to the crab’s reproductive cycle.

## Introduction

The continental margins are characterized by high temporal variability in key benthic habitat variables, (such as current regimes, sedimentation rates, and light intensity and spectral quality), with variability often related to depth [1]. In the aphotic deep sea, light intensity is often assumed to be replaced by current regimes [2] as the *zeitgeber* (i.e., the environmental synchronizer of biological rhythms) but recent data also suggest that indirect day-night synchronization may occur as a result of the presence or absence of fauna making diel vertical migrations, potentially between different depth strata within the water column [3, 4]. In general, behavioural responses of deep-sea benthic and benthopelagic fauna to variations in light, internal tides, and inertial current cycles remain poorly understood, in part because of a lack of continuous monitoring. Continuous monitoring of oceanographic variables and hourly to seasonal turnover of species compositions can permit the identification of environment drivers that shape benthic community composition [3, 5, 6].

This lack of knowledge is particularly pronounced in regions with complex topography, such as marine canyons, where detrital funneling and changing flow regimes may occur [7, 8]. This funneling and variability in flow have been identified as drivers of canyon high productivity and biodiversity [9–11]. The enhancement of primary productivity within these geomorphologies [10], concentrating zooplankton [12] and scavengers [13], contribute to an increase food availability for benthos [7] and benthopelagic species [5], as well as driving seasonal reproduction patterns of fishes, which may use canyons as breeding areas [14]. Predicting patterns of megafaunal increase of abundance in submarine canyons is of relevance for ecosystem and fishery management [15]. Although canyons may have an increased diversity and abundance in fauna when compared to adjacent continental slopes, oxygen minimum zones (OMZs), can prevent colonization by fauna less tolerant to low oxygen concentrations, hence reducing the benefits of the locally elevated food availability [16]. The response of many marine fauna to hypoxia still remains largely uncertain, so the degree to which locally low oxygen concentration bottom waters may impact species abundance and biodiversity is difficult to directly gauge [17, 18]. As a dynamic canyon system cutting through a Northeast Pacific OMZ region, Barkley Canyon (off Vancouver Island, Canada), represents an optimal study site for carrying out work aimed at improving our understanding of how deep-sea canyon communities respond to seasonal oxygen variations [19, 20]. Our study site in Barkley Canyon corresponded to the core of the North Pacific OMZ between 600–700 m and 900–1100 [21].

The section of the Barkley Canyon investigated here (850–900 m depth) is also characterized by outcropping methane hydrate deposits that mark the boundary of the temperature-pressure methane hydrate stability field [22]. The susceptibility of these gas hydrates, and their associated faunal communities with their presence to ocean warming is significant. Despite their low biodiversity, cold seeps are highly productive ecosystems [23] and are increasingly recognized as providers of ecosystem services [24]. Oxygen depletion [25], changes in water-mass circulation [26] and temperature increases [27] highlighted in future climate change scenarios represent particular threats to cold seep ecosystems in low oxygen environments, because of their potential impact on hydrate stability and worsening of hypoxia. At these cold seeps, chemosynthetic microbial production driven by oxidation of reducing substances (methane and sulphide) in discharging seafloor fluids sustains symbiont-bearing tubeworm and bivalve communities [28, 29], and free-living bacterial mats (e.g., *Beggiatoa* sp.). These in turn support higher order consumers, including fish, squid, octopii, echinoderms, other molluscs, and crabs.

The recent extension of cabled observatory technologies to the deep sea is finally permitting the continuous imaging and oceanographic monitoring required to investigate processes that shape submarine canyons and cold-seep communities [30–32] at hourly to annual time scales. A new generation of tethered mobile Internet Operated Vehicles (IOV), such as benthic crawlers, extends the potential observational footprint from a few square meters around fixed seafloor platforms [33, 34], to hundreds of square meters [35] in areas surrounding seafloor observatory nodes. This substantially extends our ability to document spatial habitat heterogeneity, and provides a larger sample size for detecting temporal variability. The crawler ‘Wally I’ is an advanced example of this mobile technology, and has been deployed in a cold-seep site in Barkley Canyon at ~890 m for several years. In addition to supporting imaging equipment, Wally I also hosts a complex suite of oceanographic sensors [35, 36].

In this study a 14 month temporally structured video-monitoring campaign to investigate the megafaunal communities of a Barkley Canyon cold-seep ecosystem was conducted, in order to test the hypotheses that (1) abundance, richness, and biodiversity changes within the surveyed area are directly driven by the oceanographic parameters such as oxygen concentration, current velocity, upwelling events and indirectly by seasonal bethopelagic and nektobenthic migrations along the canyon; (2) IOV technology can be used as a solid faunistic monitoring tool.

## Materials and methods

### The NEPTUNE network and the Barkley Canyon node

Authorization for installation of the infrastructure supporting this research was provided by the Transport Canada (www.tc.gc.ca/), after Fisheries and Oceans Canada (http://www.dfo-mpo.gc.ca/) assessed that the cabled installation would not have a negative impact on fish habitat. Field studies did not involve endangered or protected species.

**Fig 1:**
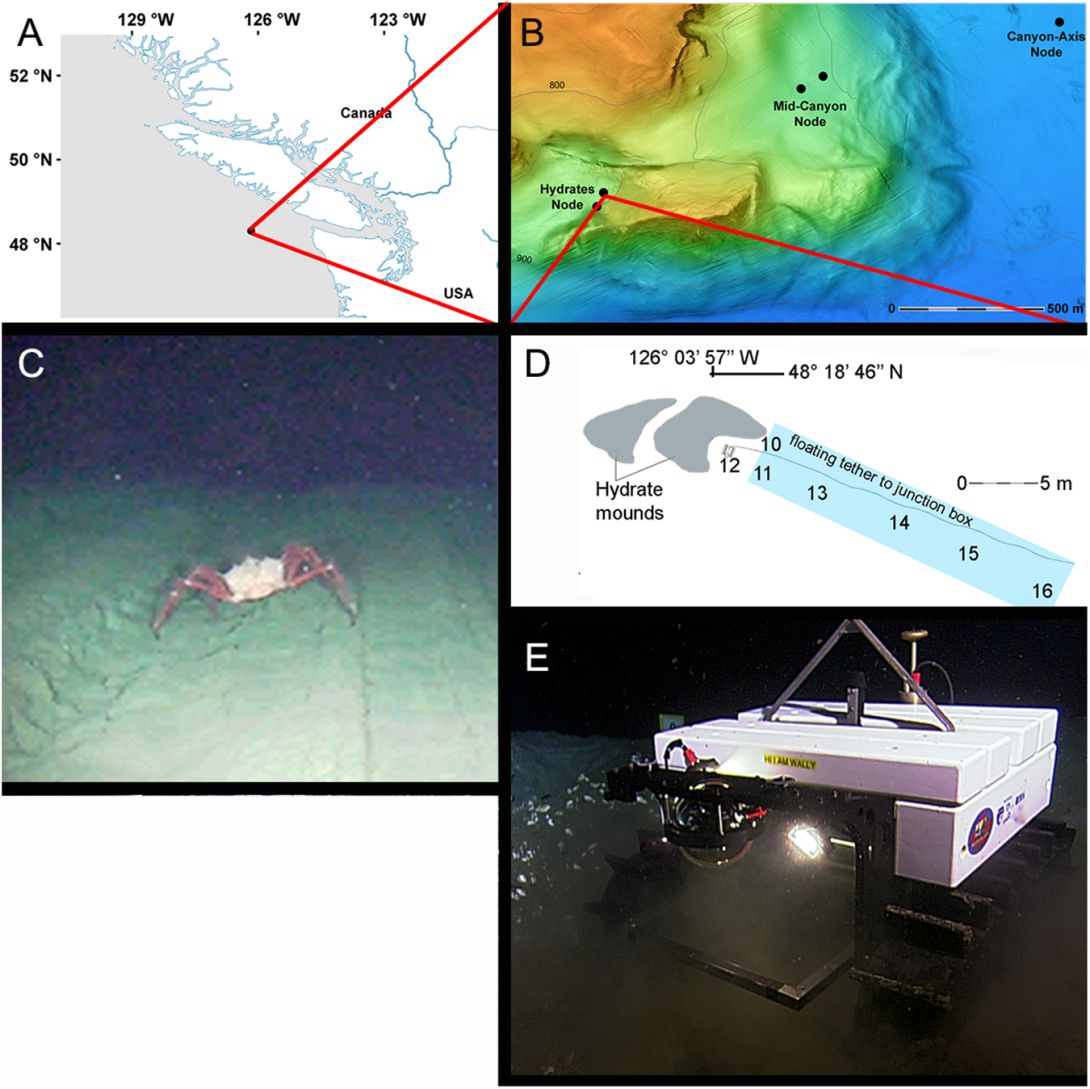
Overview map of the cold-seep site in Barkley Canyon (northeast Pacific, Canada). (A) The cabled observatory network, The Barkley Canyon node, site of Wally I crawler deployment is highlighted by a black dot. (B) High-resolution bathymetric map showing the region of Barkley Canyon investigated in the current study. The black dots within the canyon axis represent the crawler node with the nearby mid-canyon POD4 platform and other deeper nodes. (C) Image demonstrating the crawler Field Of View (FOV) as used to acquire the faunal data. (D) Map showing position of navigation waypoints arranged around the hydrate outcrop, the crawler (near waypoint no. 12 in this schematic) and the route from waypoint no. 10 to 14, representing the survey transect analysis area. The cold seep community was distributed on a soft bottom zone with no apparent emerging rocks. At the end of the transect analysis area, a 5 m depth cliff (dashed grey line) separated our study area from a from the gas-hydrate mound (E) The crawler in operation.

The NEPTUNE cabled observatory network, off Vancouver Island (BC, Canada) operated by Ocean Networks Canada (ONC; www.oceannetworks.ca) [37] supports continuous multiparametric and video observations from coastal to deep-sea habitats, providing power and data connectivity through a 840-km looped fiber-optical cable (Fig 1).

The observatory network is powered by a shore station located in Port Alberni, Vancouver Island (see Fig 1A). This sophisticated interdisciplinary monitoring infrastructure supports a node in Barkley Canyon at ~890 m depth (Fig 1B; 48°18.89′ N, 129°03.48′ W), a node which has become an important hub for *in-situ* study of the environmental drivers modulating ecosystem functioning of deep-sea cold-seep communities [38, 36]. For the duration of the study described herein, the Wally I crawler was in operation on the western flank of the main canyon, connected by an umbilical power and data cable to the node (Fig 1B). The node is also within the local Oxygen Minimum Zone (OMZ), and thus a site of further global interest as a location for ongoing monitoring during global environmental change. A full description of the study site (Fig 1C and 1D) the images recorded by the crawler (Fig 1E) installed in this area (Fig 1F) is provided in [35].

### The ‘Wally I’ crawler deployment, connectivity, operations and sensor systems

For the duration of the current study, the mobile ‘Wally I’ crawler was connected *via* a 70-m fiber-optic tether to the Barkley Canyon node [35] with syntactic foam floats arrayed every 3 m along the tether to insure buoyancy and to preclude entanglement with the crawler tracks. Crawler driving control and data acquisition were carried out in real-time *via* a custom web interface, with sensor data and imagery automatically stored to the NEPTUNE archive at the sampling resolution of each device [35].

The ‘Wally I’ crawlers’ mobility is provided by a pair of caterpillar tracks, which may be operated together, or independently to drive the vehicle forward, backward or to rotate it on the seafloor. Movement commands are made directly from the home laboratory over an internet connection.

Movement of the caterpillar tracks (and therefore, the vehicle) creates a footprint on the seafloor that could potentially disturb infauna. To prevent these impacts influencing the collected results transects aimed at collecting time series data are usually run over the same track, hence reducing the impacts of the tracks on the ecosystem. Such an operational plan was carried out in the current study (Fig 1E).

One of the primary sampling systems on the ‘Wally I’ crawler is the forward looking camera system. Video footage can be acquired using the 470 Line ROS Inspector low-light, colour camera, equipped with an 18X optical zoom whenever suitable illumination is also provided. For the current study, 20 m transects were driven every 4 hours, over the first 5 days of each month, from February 14^th^ 2013 to April 14^th^ 2014, with video data recorded on every occasion. For all transects, the camera was always oriented with a fixed tilt angle of 45° from the horizontal, from a mounting position roughly 1m from the seafloor at the front of the crawler. During operations, the crawler’s motion was kept at a near constant speed (0.02 m/s). Light was provided by two Deep-Sea Power and Light-Variable intensity lamps [35]. Lights were switched on during transect driving and turned off immediately after, in order to minimize photic contamination in the area. The total observed seafloor area recorded within each transect was approximately 60 m^2^.

The ‘Wally I’ crawler can mount a plethora of sensor systems, and for the duration of the current study an upward facing 2-MHz ADCP (Nortek Aquadopp Profiler AQD 9917), measuring water velocity and temperature at 1 mab and a Seapoint Turbidity Meter, measuring at approx. 0.2–0.3 mab the turbidity were equipped. An adjacent observatory (the POD4 NEPTUNE node), situated at a distance of ~500 m from the site of crawler operations, within the mid-canyon at 896 m depth (48°18.8923′ N, 126°03.4804′W; see Fig 1B) was simultaneously recording pressure, water density data and oxygen, as collected respectively by a CTD (Sea-Bird SeaCAT SBE16plus V2 7027) and an oxygen sensor (Sea-Bird SBE 63 630111). Collected data at 1 min frequency throughout the entire survey period, both at times coinciding to the collection of video transect data as well as during the periods of immobility between crawler transects. These data are freely available online (http://dmas.uvic.ca/DataSearch). Additionally, the Bakun Index, as a proxy of upwelling and downwelling processes, was also computed at 48N, 125W from National Oceanic and Atmospheric Administration *(*NOAA) / National Marine Fisheries Service (NMFS) / Pacific Fisheries Environmental Group (PFEG) data.

### Faunal data collection and treatment

Following collection of the transect video data, one user made visual counts of the megafauna individuals present within each transect. The same user analysed all collected videos, identifying individuals to the lowest taxonomic level as possible using the NEPTUNE Canada Fauna Identification Guide [39]. All video recorded were archived and are available online through the Oceans 2.0 portal (http://dmas.uvic.ca/DataSearch).

Although a near constant crawler speed and camera angle were maintained throughout the duration of the study, occasional variabilities in field of view recorded did result from small differences in seafloor relief across the transect survey length. To account for this, we analysed only the portions of video data where 75% of the field of view encompassed the seabed, and with sufficient water transparency for animal classification and counting.

Slight variabilities in transect duration also occurred, due to slight differences in seafloor angle, firmness and due to technical driving and internet connectivity issues (e.g. interruption of signal send / return from our working office in Barcelona to the Pacific). Therefore, to make the biological data recorded as comparable as possible over the video transects collected, we standardized visual counts for the each megafaunal taxa and transect to the maximum video duration recorded within the study, which was 23 min.

In order to highlight species count patterns as proxies of seasonality, we plotted time series of average visual counts (±sd) for each month, based on all transects recorded during the 5 days of monitoring at the start of each calendar month throughout the 14 month period. In the resulting plots, we superimposed horizontal dashed lines representing the ‘Midline Estimated Statistic of Rhythm (MESOR; [4])’, computed by re-averaging all monthly values. Their overlaying onto the monthly plot allows the identification of significant seasonal increases or decreases in abundance.

Current velocity component-vectors (i.e. North-South and East-West, xy direction data collected via the ADCP system) were transformed to flow magnitude (m/s). All environmental data collected by the crawler and the POD4 node (see 2.2) were averaged into 4 h bins to match the video-sampling frequency. Bakun Index values, the daily-averages of wind-driven cross-shore transport, were computed from Fleet Numerical and Meteorology Oceanographic Centered (FNMOC) 6-hourly surface pressure analyses.

### Analysis of the community structure linkage with oceanographic variables

A Kruskal Wallis test was carried out using the R statistical language [40], to detect significant differences in the number of megafaunal visual counts (of each species or taxa logged) between months, following the methodology presented in [41].

A Nonmetric Multidimensional Scaling (NMDS; [42]) analysis was performed in the R library *vegan* [43] to visualize the level of similarity among species presence and visual counts (i.e. assemblage structure) together with correlated environmental vectors into a Cartesian plane. The Bray-Curtis dissimilarity Index was used to quantify the dissimilarity between megafaunal species based on the time series visual counts, while a Wisconsin double standardization was performed (since this improves the gradient detection ability of dissimilarity indices; [44]). The significance of the estimated determination coefficients (r2) of the environmental variables fitting onto the species ordination, produced by the NMDS analysis, was estimated using a permutation test. Results of the species ordination using NMDS and the correlation with the environmental variables are shown in a plot, with environmental vectors (arrows) showing significant (p<0.05) maximum correlation with the species ordination. Results are shown only for the environmental variables significantly correlated to the species ordination.

Biodiversity was calculated with two indices presented as mean diversity per month: the Shannon index (H’) and the Simpson Index, presented as 1-D. In a similar manner, we presented the species richness (S). The monthly abundance (number of individuals of all species) was normalized to the maximum number of transects per month (i.e. 48). Herein we refer to these means as biodiversity indices, Shannon index, Simpson Index or abundance as appropriate.

### Ethological remarks

Behavioural observations were logged for the motile fauna imaged during the crawler surveys. These, though opportunistic, were described in order to increase general information on the ethology of several deep-sea species, and may represent pivotal aspects of the functioning of some ecosystems, particularly in terms of inter- and intraspecific relationships [45], and also to help support the second hypothesis of the current study, that IOV technology has application as a solid faunistic monitoring tool.

## Results

438 video transects, representing a total of 92 h of footage of acceptable quality for analysis was collected during the 14 month monitoring period. No video data was collected during March 2014 due technical problems. 7698 megafauna individuals, belonging to 6 phyla were observed (S1 Table). These included representatives of 26 taxa, 12 of which could be identified to species level, with remaining individuals classified to higher taxonomical levels (S1 Fig).

We detected considerable differences in megafaunal abundances, between species and temporally, throughout the study period (see S1 Table). The most abundant species within the study period were the sablefish (*Anoplopoma fimbria*) with 2214 visual counts, representing 29% of the total observed megafauna. Buccinids (Buccinoidea) were also abundant with 1318 individuals representing 17% of the total megafauna. The third most abundant taxon was hagfish (*Eptatretus stoutii*; i.e. 852, as 11%). The fourth was abundant was Scyphomedusa (*Poralia rufescens*) with 789 individuals (10%). Next were rockfish (i.e. from the family Sebastidae) with 573 filmed individuals (7%) and small crabs (of undefined taxon) showing a similar abundance (N = 535, 7%). The final significantly abundant taxon, with 449 records, was ctenophores (*Bolinopsis infundibulum*), representing 6% of recordings of fauna.

### Seasonal patterns

**Fig 2:**
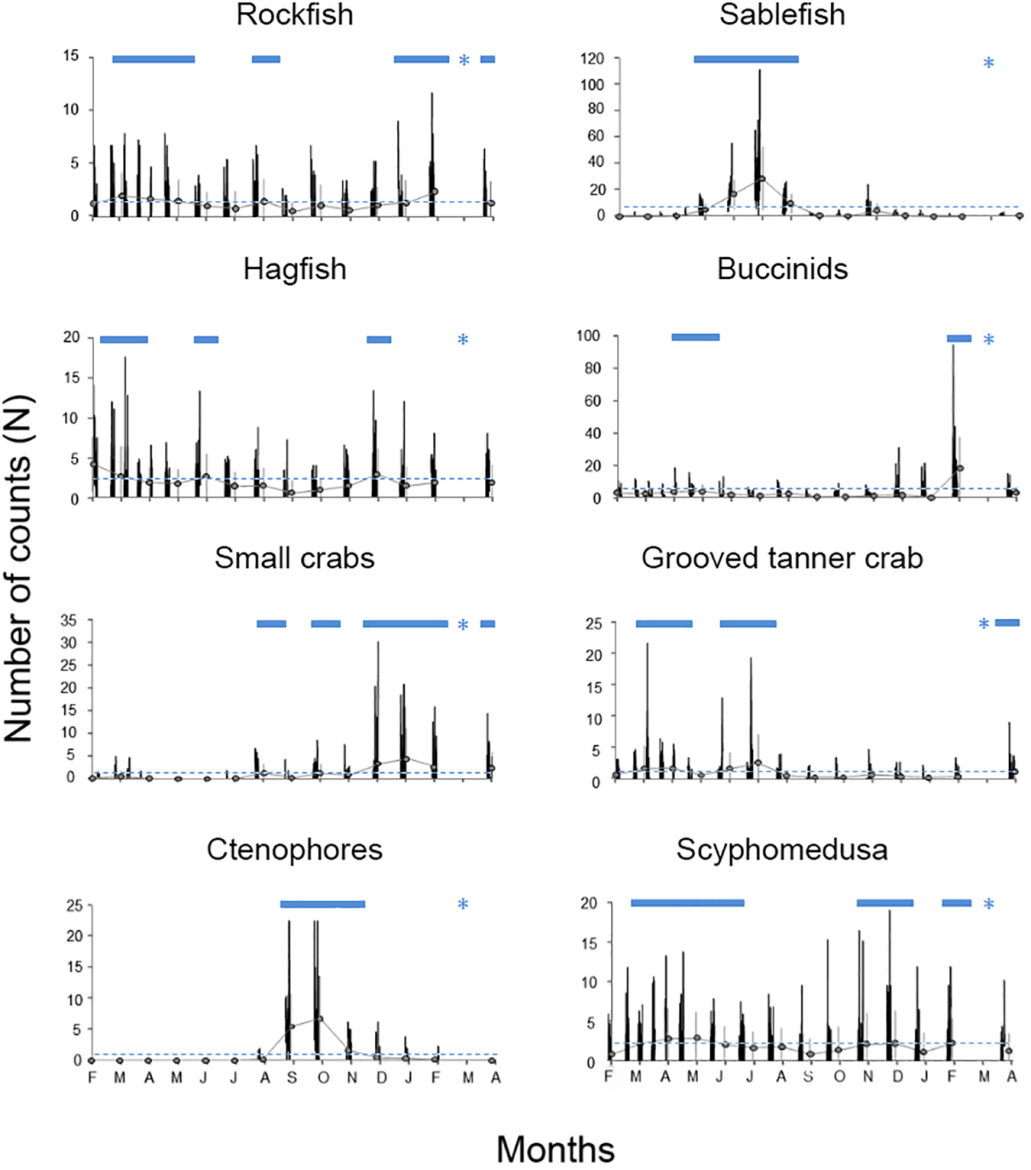
Visual counts time series (i.e. from 14^th^ February 2013 to 14^th^ April 2014) for the 7 most abundant megafaunal species and the particular case of the grooved tanner crab, as reported by crawler video-imaging. Average values (the grey dots) have been reported in order to better highlight the occurrence of seasonal trends. From left to right and from top to bottom taxonomic units are: Rockfish (i.e. Sebastidae); Sablefish (*Anoplopoma fimbria*); Hagfish (*Eptatretus stoutii*); Buccinid (*Neptunidae*); Small crab; Grooved tanner crab (*Chionnecetes tanneri*); Ctenophore (*Bolinopsis infundibulum*); and finally, Scyphomedusa (*Poralia rufescens*). Horizontal blue dashed lines correspond to the Midline Estimated Statistic of Rhythm (MESOR) and horizontal blue lines identify significant seasonal increases visual counts increases. Blue asterisks correspond to a month with no data due technical problems.

Time series analysis was carried out for the 7 most abundant megafaunal species (Fig 2, plotted to show the occurrence of any monthly variation as a proxy of seasonal fluctuation). Grooved tanner crab count time series were also plotted to show the particular variabilities in their abundance resulting from their reproduction dynamics. Since no video data was collected during March 2014, we assumed significant values during this month when the concomitant months were significant. An overview of the time series analysis showed species peaking frequently from February to August 2013 and from January to April 2014. A general lack of species occurred during the central months of the study (autumn and December 2013).

A close-up of Fig 2 showed that rockfish were present in roughly consistent densities throughout the observation period, though significantly higher (i.e. above the MESOR) numbers of them were observed during spring and August 2013 and from January to April 2014. We observed a significant sharp peak in sablefish visual counts during late spring (March) and summer 2013 reaching their maximum abundance in July, with a mean of 29.1±23.8 SD individuals per transect. Hagfish were also uniformly abundant throughout the study, with a modest but significant count peak in February-March and July 2013, and another one of similar magnitude in January 2014. Buccinids showed low but consistent abundances during much of the survey period with a mean of 3.3±4.46 SD individuals per transect. Though a moderate but significant increase occurred from May to June 2013 and a sharper one occurred in February 2013 (i.e. 18.3±18.9 SD individuals per transect).

Small crabs were rarely present during the study period, although their numbers were reaching significant levels intermittently from August 2013 to April 2014 (i.e. August, October and from December 2013 to April 2014), reaching a maximum in January (i.e. 4.5±6.7 SD individuals per transect). Grooved tanner crabs were present all year-round with visual counts increasing moderately but significantly during March and April 2013 (1.6±3.7 and 1.6±2.1 SD individual per transect, respectively) and again in April 2014, with 1.1±2 SD individuals per transect being observed (coinciding with reproduction; see the Behavioural Remarks Section below). Another significant peak was observed during early and mid-summer 2013 (June and July).

Ctenophore visual counts displayed clear seasonal patterning. A sharp increase in numbers occurred during autumn with a maximum density observed in October 2013 (i.e. mean density of 6.9±6.4 SD individuals per transect). Finally, the Scyphomedusa was significantly present during almost all months of the monitoring period (i.e. from February to June and from November to December 2013 and February 2014) with a moderate count decrease from mid-summer to mid-autumn.

**Fig 3:**
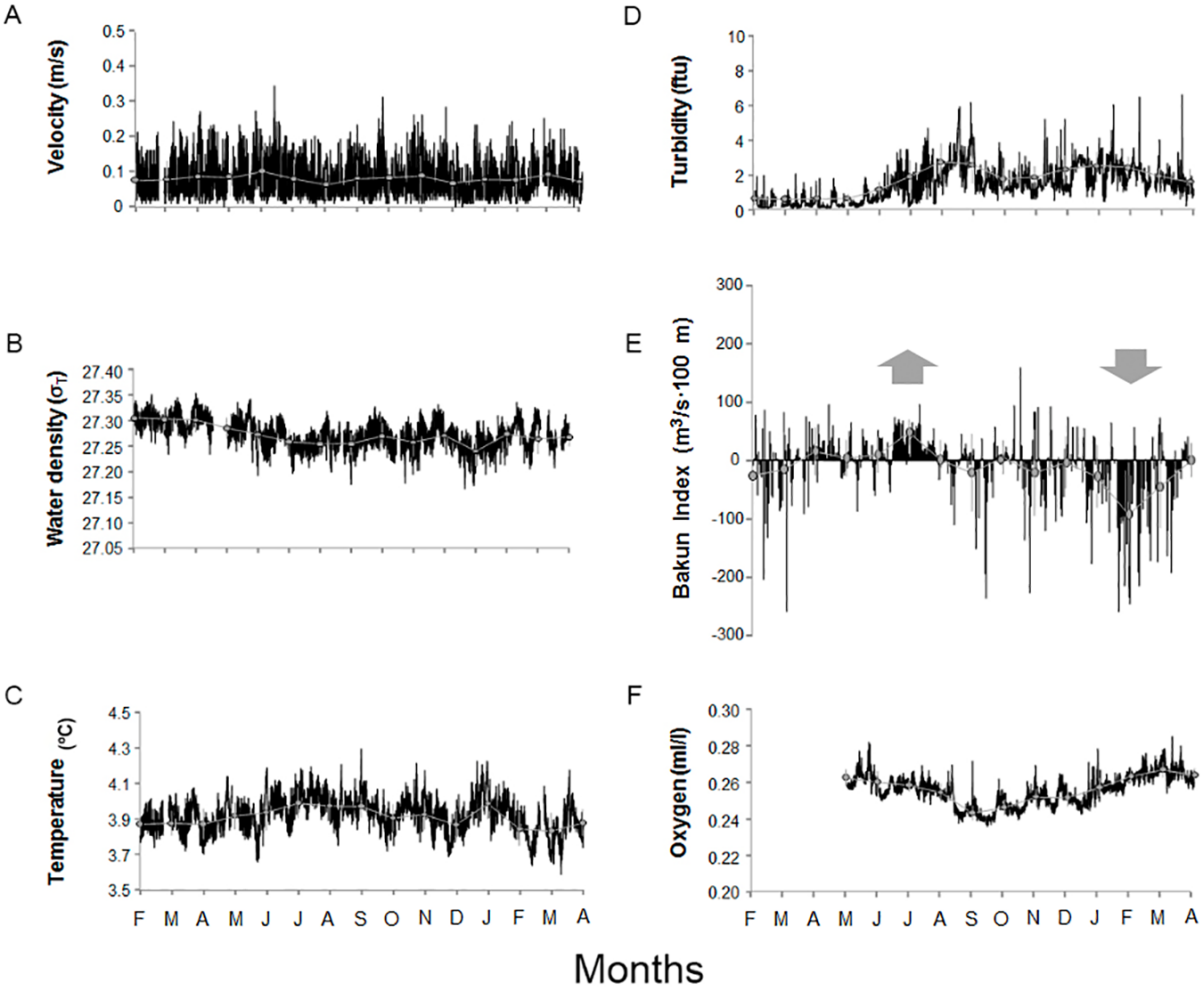
Oceanographic parameters time series (i.e. from 14^th^ February 2013 to 14^th^ April 2014), as recorded by crawler and the nearby POD4 platform within the Barkley Canyon. Average values (the grey dots) have been reported in order to better highlight the occurrence of seasonal trends. Gaps in data acquisition were due to instrument malfunctioning or downtime for the cabled infrastructure. These parameters are: (A) Velocity; (B) Water Density; (C) Temperature; (D) Turbidity; (E) Bakun Index; and finally, (F) Oxygen. We used up-looking and down-looking arrows to highlight upwelling and downwelling periods respectively.

Fig 3 shows the time series data for the investigated oceanographic parameters, as measured by the crawler on board sensors and the nearby POD4 network node. There were a few occasional gaps in data acquisition produced by sensor malfunctions or data network issues. A patterning in seasonality is evident in water density, temperature and turbidity measurements. Additionally, the Bakun Index and oxygen concentrations measured during the 14 months of the study showed seasonality in the values measured. Velocity was highly related to the diurnal and semi-diurnal tidal cycle. The Bakun Index presented the lower values during February 2013, indicating predominant downwelling processes ongoing during that month. A progressive increase in the Bakun Index led to positive values as sustained pulses (i.e. upwelling events) from mid-April to August 2013. Coinciding with these upwelling events, we detected the greatest recorded variations in turbidity. Elevated turbidity levels were coincident with the first shallow water incursion in the local deep-sea area in August, which brought in warmer and less salty waters. Oxygen levels ranged from 0.24 (October 2013) to 0.29 ml/l (March 2014), with a mean of 0.26 ml/l. Oxygen concentrations gradually decreased in August, reaching their minimum values in September-October. Thereafter, oxygen progressively increased to return to the initial concentrations by the end of the study. This lower oxygen period coincides in time with Bakun Index reaching values close to zero. Water density also showed a weak seasonal trend, with generally warmer and fresher water masses present at the study site from July to October.

### Analysis of the community structure linkage with oceanographic variables

Kruskal-Wallis tests (Table 1) revealed significant differences among months for the most abundant species, with a *p*-values ≤ 0.01.

**Table 1:** Kruskal-Wallis test among the 14 months of the study for the 8 most abundant species with 13 degrees of freedom.

Taxonomical Units	Common name	x^2^	*p*-value
Sebastidae	Rockfish	29.23	< 0.01
*Anoplopoma fimbria*	Sablefish	346.80	<0.01e^-15^
*Eptatretus stoutii*	Hagfish	44.39	<0.01e^-4^
Buccinoidea	Buccinids	275.13	<0.01e^-15^
	Small crabs	99.50	<0.01e^-15^
*Chionoecetes tanneri*	Grooved tanner crab	46.81	<0.01e^-4^
*Bolinopsis infundibulum*	Ctenophores	275.13	<0.01e-^15^
*Poralia rufescens*	Scyphomedusa	27.30	<0.01

**Fig 4:**
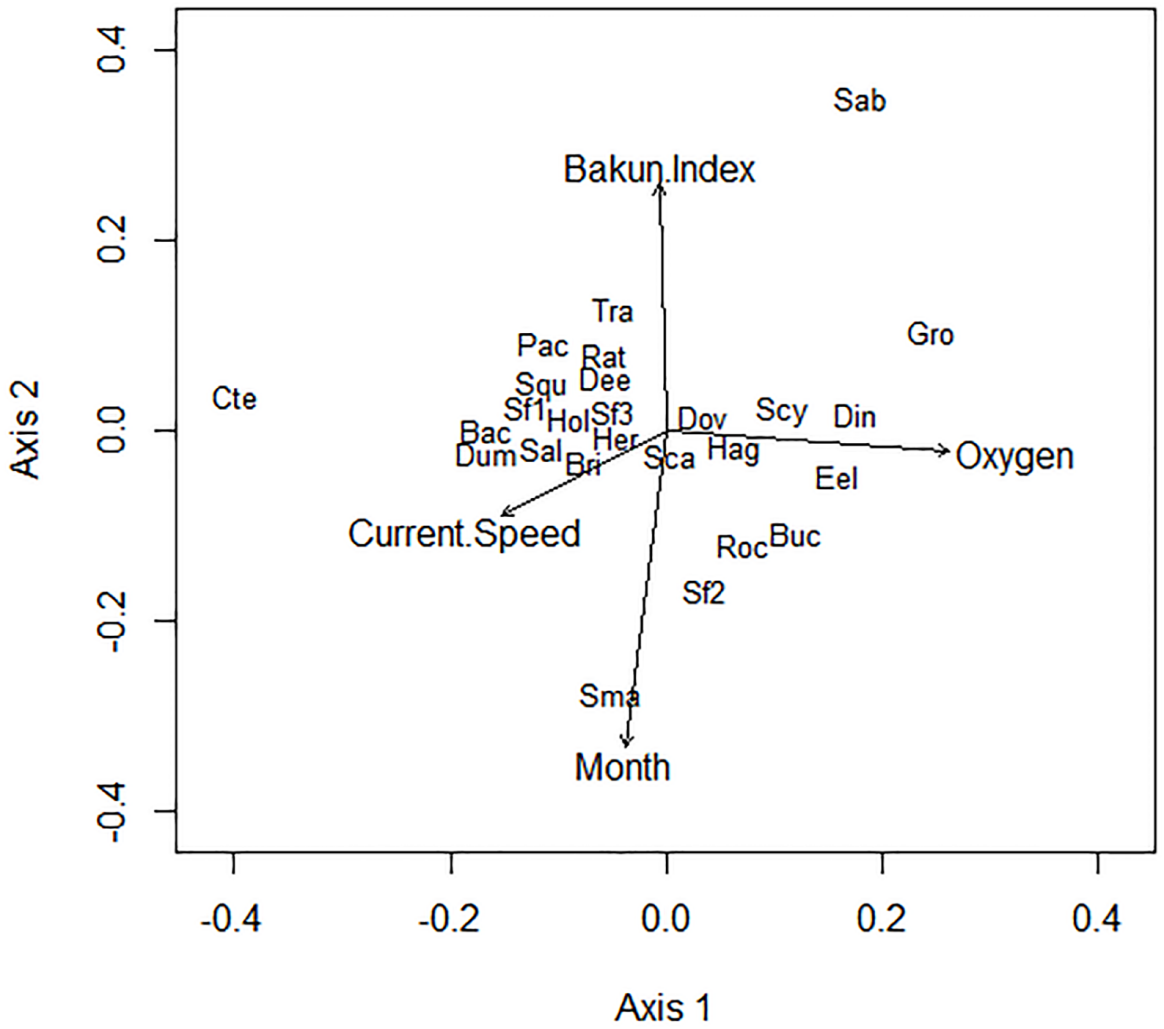
Results of fitting environmental variables onto species ordination. **The increasing gradient of the environmental variable is indicated by the vector direction. The vector length is proportional to the correlation between the variable and the ordination pattern of the species.** Species abbreviations are (taxonomical order; see S1 Table): Eel (Eelpout, *Licenchelys* spp.); Dov (Dover sole, *Microstomus pacificus*); Dee (Deep sea sole, *Embassichthys bathybius*); Pac (Pacific halibut, *Hippoglossus stenolepis*); Rat (Rattail, Coryphaenoides spp.); Roc (Rockfish, i.e. Sebastidae); Bac (Blackfin poacher, *Bathyagonus nigripinnis*); Sab (Sablefish, *Anoplopoma fimbria*); Hag (Hagfish *Eptatretus stoutii)*; Sal (Salp, Salpidae); Dum (Dumbo octopus, *Grimpoteuthis* sp.); Squ (Squid *Gonapus* sp.); Buc (Buccinids, Neptunidae); Bri (Brittle star, Ophiuroidea); Sf1 (Starfish, Asteroidea); Sf2 (Starfish, Zoroasteridae); St3 (*Hippasteria* sp.); Hol (Holoturian, Holoturoidea); Her (Hermit crab, Diogenidae); Sca (Scarlet king crab, *Lithodes couesi*); Gro (Grooved tanner crab, *Chionnecetes tanneri*); Sma (Small crabs); Cte (Ctenophore, *Bolinopsis infundibulum*); Scy (Scyphomedusa, *Poralia rufescens*); Din (Dinner plate jelly, *Solmissus* sp.); Tra (Traquimedusa, *Voragonema pedunculata*).

Environmental parameter fitting onto species ordination as obtained from NMDS analysis is shown in Fig 4. Current velocity, Bakun Index, dissolved oxygen and months were the ‘environmental’ variables showing significant (p<0.05) maximum correlations with the species ordination, with r2 values of 0.14, 0.29, 0.29, and 0.47 respectively. Month was included as a proxy for the presence of a seasonal pattern in abundance variations (See Fig 2 and S1 Table for species individual counts). The Bakun Index was negatively correlated with the variable month (Fig 4) likely resulting from the progressive change from predominantly upwelling to downwelling conditions over the monitoring period (See Fig 3). A weaker negative correlation was also observed between dissolved oxygen concentration and current velocity (Fig 4). An inverse correlation between rockfish visual counts and Bakun Index values was also indicated (i.e. increases in rockfish abundances were correlated with downwelling), whereas sablefish, correlated with both Bakun Index and low current velocities. As with rockfish, elevated densities of buccinids correlated with periods of sustained downwelling (i.e. negative Bakun Index values), occurring toward the end of the monitoring period. A positive correlation with increasing oxygen was also indicated for the buccinids. The similarities in the abundance curves of buccinids and rockfish is shown in Fig 4. In general, grooved tanner crabs were observed in highest abundances during the first half of the study (from February to August 2013), with these higher abundances correlating with higher oxygen levels and lower current velocities. Small crabs’ abundance was clearly higher during the second third of the study (Fig 4). In the case of ctenophores, NMDS showed that highest abundances correlated with lower oxygen levels. The abundance of another cnidarian, the Scyphomedusa, was strongly associated with periods of weak current flow and higher oxygen concentrations.

**Fig 5:**
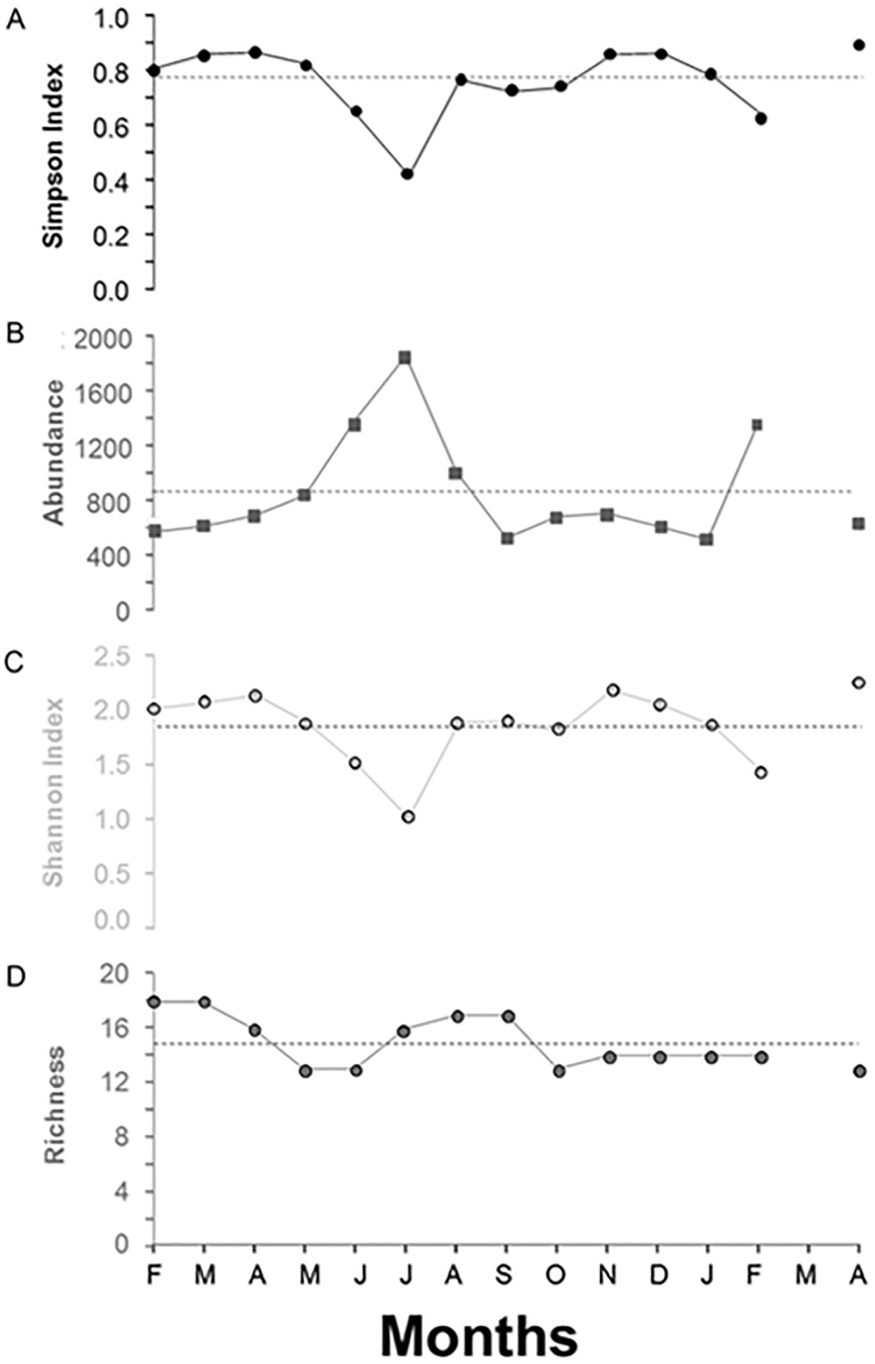
Biodiversity, richness and megafaunal species visual counts (i.e. from 14^th^ February 2013 to 14^th^ April 2014) time series, as reported by crawler video-imaging. Biodiversity have been reported as mean per month Shannon Index (C) and Simpson Index (A). Similarly, we reported the species richness (D). Abundance (B) was normalized for the maximum transects of the study period (i.e. 48). Horizontal dashed lines correspond to the Midline Estimated Statistic of Rhythm (MESOR).

Species richness was almost constant throughout the studied period (14.93 ±1.82 species/60m^2^) with two moderate drops, one from March to June 2013 (13 species/60m^2^) and another one of the same magnitude in October of the same year with similar levels lasting until the end of the study (Fig 5D). Abundances increased progressively from low levels at the beginning of the study (550.42 individuals/60m^2^ in February 2013) being in March 2013 very close to the significant level (MESOR; 814.05 individuals/60m^2^). A significant peak occurred from June to August 2013 (maximum 1810.56 individuals/60m^2^ in July) and again in February 2014 (1412.59 individuals/60m^2^; Fig 5B). The two biodiversity indices values (H’ and 1-D), respectively Fig 5C and 5A, followed a very similar pattern. Both were relatively constant (H’: 1.83 ±0.34; 1-D: 0.74±0.13) apart from 3 decreases below the significant levels leading to low points. June to July (minimum being in July: 1.00), October 2013 (1.79) for H’ and June to July (minimum being in July: 0.40) and September to October 2013 (minimum being in September: 0.71) for 1-D. Both indices experienced a drop in February 2014 (H’: 1.36; 1-D: 0.59).

### Ethological remarks

Ethological observations were made for a range of different megafaunal species (see S2 Table for dates and hours, S1 Fig for a reference on their physical appearance and S1 Table for their total visual counts). Fishes were usually observed lying on the seabed, while ignoring the approaching crawler. Some species (e.g. Dover sole, *Microstomus pacificus*, Deep sea sole, *Embassichthys bathybius*, Pacific halibut, *Hippoglossus stenolepis*, rockfish and hagfish) retreated approximately 2 to 3 m when crawler approached too close If they were lying directly in front of the crawler. Rattails (*Coryphaenoides* spp.) and Blackfin poacher (*Bathyagonus nigripinnis*) did not react at all to the crawler approach.

We observed once the agonistic display of a rockfish toward the crawler, which consisted in it approaching very close to the camera with its mouth open and then escaping (S2A Table). Eelpouts were also seen escaping, by touching the seafloor and producing mud puffs, quickly leaving the vicinity of the crawler. Sablefish approached the camera of the crawler and then swam away from the field of view. During one encounter, a sablefish swam close to crawler for approximately 10 m of the transect (S2B Table). On most occasions, sablefish appeared actively swimming but in some occasions they were observed lying on the seabed. Hagfish were similarly observed either laying on the seafloor or actively swimming but never drifting.

Dumbo octopii (*Grimpoteuthis* sp.) were observed lying on the seafloor on four occasions, without reacting to the presence of the crawler. In contrast, another cephalopod (the squid *Gonapus* sp.) quickly swam away releasing ink when it encountered the approaching crawler.

Feeding agonistic behaviour between grooved tanner crab and scarlet king crab (*Lithodes couesi*) was observed (S2C Table). While a male scarlet king crab was trying to open a *Calyptogena* spp. clam, a grooved tanner crab appeared within the frame and tried to steal its prey (i.e. by advancing its chelipeds toward the clam). This interaction took 8 minutes, during which the scarlet king crab responded by hiding the prey below its body.

Grooved tanner crabs were particularly undisturbed by the presence of the crawler walking very slowly upon encounter. Only one individual displayed an agonistic posture (S2D Table). Several observations on tanner crab reproductive behaviour were made in March and April 2013, and again in April 2014. Male and females were observed facing each other, touching their respective maxillipeds whilst the male took both female claws. The couples either ignored the crawler or avoided it. In the latter case, the males (considerably bigger than the female) lifted his partner by one claw above its body and walked away from the crawler, to later restart the mating (S2E Table). Finally, we detected on 5 occasions females carrying eggs from March to April 2013 (S2F Table).

## Discussion

This study represents the first, high resolution documentation of seasonal patterns in megafaunal abundances and biodiversity at a cold-seep site. Our results of the visual counts of individuals observed across a 60 m^2^ area of seafloor, made throughout a 14 month period, provide some indications of abundance patterns for different species through consecutive months, as a proxy of seasonality in behaviour and recruitment. Year-long monitoring programs have been used successfully in other studies to document seasonality in deep-sea megafaunal abundances (see [46–47]. Similar species abundance levels at the beginning and end of our time series may indicate annual patterns. We observed individuals belonging to 6 phyla as being particularly abundant (26 taxa within cnidarians, ctenophores, arthropods, echinoderms, molluscs, and chordates). Our results are comparable with those from two other studies conducted at fixed observatory nodes in nearby non-seep regions of Barkley Canyon (see [33] and [48]). Slight differences in visual counts and composition of the megafaunal communities between these sites may be attributed either to the different methodological approaches (i.e., fixed *versu*s mobile observations), to differences in topography and oceanographic conditions occurring along the depth gradient [33, 5, 18] or with proximity to the canyon walls.

Compared with imaging from a fixed camera platform, running imaging transects with the crawler provided a larger sample of the less abundant, benthic and benthopelagic megafauna that inhabited the seafloor and adjacent benthic boundary layer [22]. Nevertheless, we underline the necessity of combining video transects (as performed in the current study) with close-up imaging of the seabed for detecting and identifying very small species (e.g., Caridea, Cirripeda or *Heptacarpus* sp.) as well as buccinids and small crab species), that are difficult to image from a mobile platform.

### Seasonal patterns and the oceanographic influences

We detected monthly patterns in abundances of 8 benthic megafaunal taxa (rockfish, sablefish, hagfish, buccinids, small crabs, grooved tanner crab, ctenophores and Scyphomedusa) as shown by the time series analysis, the Kruskal-Wallis test and the NMDS analysis. Monthly abundance patterns can be considered a proxy of local seasonal drivers of species abundances [34]. Massive seasonal bathymetric displacements of benthic or benthopelagic megafauna on continental slopes can be linked to food availability [49–51], growth, and reproduction cycles (e.g. [46, 52]), as well as to changes in predation pressure [53]. In high latitude biogeographic regions such as the NE Pacific, which display strong seasonality in primary production [54], such seasonal displacements may serve as integral energy pulses that support the trophic web [55].

Conversely, less information exists on deep-sea populations’ responses to seasonal oceanographic variations, especially where benthic habitats occur within OMZs (i.e. depth strata with oxygen concentrations < 0.5 ml/l; [56]). The depth of our study site corresponds to the core of the OMZ in the North Pacific [33]. The high-frequency variations in water mass physical properties (i.e., water density, O_2_ levels, current velocity and temperature) detected at this cold-seep site were relatively minor in comparison with those measured at shallower depths of the canyon in [33]. This suggests that the region surveyed by the crawler in the current study, at ~890 m depth, was below the depths affected by shelf-edge upwelling, as observed at similar depths. Our results in the seasonal patterns in abundances, as well as the NMDS analyses suggest that some of our assessed species are indirectly influenced by upwelling (sablefish) and downwelling (rockfish and buccinids). In [33], the authors suggested that an inverse correlation of dissolved oxygen concentration and temperature could be an indication of water mass changes, as seems also to have been the case in the area investigated in the current study. Sablefish day-night nektobenthic migrations in Barkley Canyon are in antiphase with current speed, a possible compromise strategy between search for prey and energy saving due to physiological limitations [5, 22]. Our data suggest that sablefish could follow a similar strategy also at a seasonal scale with animals avoiding shallower depths affected by upwelling. This would explain the peak in sablefish visual counts, the peak in total megafaunal abundance and the sharp decrease of biodiversity in July. Significant seasonality in sablefish abundance has been previously reported in SE Alaska, with the local population associated with deeper waters during the winter period [55].

Buccinids exhibited a sharp increase in densities during February 2014. These gastropods are both predators and scavengers, known to form aggregations either to exploit prey [45, 57] or to mate [58]. They were not affected by currents above the seafloor, yet their maximum abundances coincided with the most pronounced downwelling period of the year. NMDS showed a positive correlation of buccinid abundances with increasing oxygen levels. In contrast, gastropods are known to be particularly resistant to hypoxia [59]. This suggests an indirect effect of downwelling on the abundance of this species in our location. In agreement with this, [48] found that *B*. *viridum* migrated from shallower depths to a location ~200 m from our study area, presumably to avoid enhanced currents. Highest rockfish densities also coincided with the strongest downwelling period (from January to April 2014). The NMDS indicated that their abundances were affected by the same environmental factors that influenced buccinid abundances. Although rockfish has often observed to be resident at fixed locations [60–62], our findings suggest that at least some individuals move into this area to avoid enhanced currents in a similar strategy to the buccinids. This hypothesis needs to be confirmed with further studies. Although buccinids and rockfish were also observed outside of the strongest downwelling periods, their numbers were close to the significant threshold and coincided with Bakun Index values close to zero. Additionally, rockfish are the major contributors to sablefish diet [63]. This could explain the decrease in rockfish counts during June and July when sablefish abundances were the highest of the study period.

Hagfish and the Scyphomedusa increased in abundance coinciding with an increase of oxygen concentration following the period of low oxygen concentrations from August 2013 to January 2014. NMDS outputs suggest that these species have their ideal oxygen threshold above the minimum levels observed in our study. Data on small crab abundance suggest a more complicated picture, with both seasonal (month-scale) fluctuations and diel trends negatively correlated with oxygen levels [22].

A seasonally-related change in the depth zonation of the grooved tanner crab has been reported for the US West coast, with local densities at particular depths varying temporally and as a consequence of their growth and reproductive cycle [64]. Sexes are also completely segregated by depth during spring and summer, whereas males and females mix in autumn and winter months, when males move into deeper waters [65]. The observed peaks in visual counts of individuals within the current study, during March-April and June-July 2013, together with the subsequent sharp decrease and maintenance of low abundance maintained until the next spring, could be related to a similar seasonal displacement. Here, high visual counts of small crabs were made in December 2013, with abundances remaining high throughout winter. To our knowledge, there have been no previous studies of seasonal recruitment of *Chionoecetes tanneri*, so this potential evidence of a synchronous recruitment event should be considered with caution. According to [66] female Tanner crabs, which live at deeper water depths than males, move shallower for egg release and mating in March and April. Their higher abundance here in March-April, during higher oxygen levels, could be explained by a requirement of egg-carrying females to reduce the energy investment for oxygenating their eggs, as has been observed for other crustaceans [67]. To summarize, abundance trends and ethological observations throughout the study period, suggest that a massive reproductive aggregation of grooved tanner crab occurred during March and April 2013, following a previous migration from shallower waters (males) and deeper waters (females). This may have resulted in an associated peak in recruits observed in from December 2013 to February 2014.

Ctenophores were present during the autumn months of 2013. In [33], the authors also detected an increase in ctenophore visual counts in October-December at a nearby mid-canyon site. After occupying shallower depths near the surface ocean in spring and summer, a period coinciding with the maximum surface chlorophyll levels, [68] found larger ctenophore individuals overwintering in deep waters, where they prey on copepods. Such vertical displacement of larger zooplankton represents a trade-off between feeding efficiency and predation avoidance [69]. Ctenophores are able to perform vertical movements at various temporal scales (i.e., diel, tidal, and seasonal migrations) thanks to their high tolerance of transient hypoxia [70]. Our time series counts and NMDS outputs support the occurrence of a similar phenomenon at the cold-seep site investigated here. We detected a subsequent increase in the visual counts of the Scyphomedusa, a species previously reported as being associated with OMZs [71]. Although little is known about the diet preferences of the species, other Scyphomedusa prey on *Bolinopsis infundibulum*, which could explain the concurrent peaks of these two gelatinous species [72, 68]. The same occurrence pattern for this jellyfish has been also observed in the mid-Barkley Canyon [33]. As previously mentioned, this species was not present in video-data during the periods with the lowest oxygen levels, likely due to physiological limitation.

Although community composition differed throughout the study period, changes in species richness did not reach an order of magnitude, with a similar number of species replacing some of the previously existing ones. This was also reflected in the two biodiversity indices, which maintained a relatively constant level apart from 3 sharp decreases leading to low points (i.e. June-July, September-October 2013 and February 2014). In these particular periods, a single species (i.e. sablefish, ctenophores and buccinids, respectively) was disproportionally abundant, resulting in low biodiversity through low evenness. Taken together, these findings confirm our first hypothesis that abundance, richness, and biodiversity annual changes in relation to oceanographic conditions are directly driven by oxygen, current velocity, and upwelling/downwelling, and indirectly driven by seasonal benthopelagic and nektobenthic migrations along the canyon.

### Methodological remarks

Crawler motion produced a transient sediment clouds in the water column behind the vehicle. Since all monitoring and environmental sensors were mounted at the front of the crawler, this is not expected to have affected any of the data values measured by the sensors. Though capable of surveying similar areas of seafloor as ROVs, crawlers (particularly IOVs such as ‘Wally I’) do not need ship assistance to operate, and in this study a crawler was efficiently used to perform a long-duration, spatially extensive study. In addition, ROVs are commonly perceived as “foreign objects” to the local or periodically visiting fauna, whereas permanently deployed, slower moving IOVs are permanent elements of the local benthic panorama rapidly accepted by the resident fauna. Crawlers may therefore be considered a less intrusive ethological monitoring technology (e.g., for monitoring reproduction and making natural situation feeding and agonistic behaviour observations), since they are likely “accepted” by the local fauna.

The disturbance caused by crawler lights on the behaviour of resident animals has not been well investigated. Sablefish have been previously found to be attracted by observatory lights for short periods of time and then to leave the field of view of cameras mounted on the NEPTUNE nodes [5]. This short-duration light attraction suggests that animals are unlikely follow the crawler during transects and therefore unlikely to be recounted erroneously as successively imaged individuals. The remainder of the mobile species detected didn’t react or showed only minor reactions to crawler presence, avoiding it only when it was in extremely close proximity. In any case, bias generated by the presence of the crawler would have been maintained as constant throughout the study period given the uniform methodology employed in collecting the transect video data, and therefore not prevent the detection of seasonal patterns in megafauna abundance or biodiversity, or the successful collection of ethological observations. Therefore, our second hypothesis, that IOV technology represents a reliable faunal monitoring tool for work in the deep sea is also confirmed.

## Supporting information

S1 Table Monthly total visual counts and percentages for the different megafaunal species studied during the 14 months of video acquisition. (TIF)

S2 Table Date and time of the reported ethological remarks. (A) Rockfish (Sebatidae) agonistic display against the crawler (i.e. approaching the camera with the open mouth and then escaped). (B) Sablefish (*Anoplopoma fimbria*) swimming close to the crawler. (C) Male scarlet king crab (*Lithodes couesi*) feeding behavior and agonistic interaction with a grooved tanner crab. (D) Grooved tanner crab (*Chionnecetes tanneri*) agonistic display against the (i.e. an elevated body posture and chelipeds forward projection). (E) Grooved tanner crab reproduction behaviour. (F) Female grooved tanner crabs carrying eggs.
(TIF)

S3 Table Visual counts of studied megafaunal species together with concomitant environmental data averaged at 4 h frequency. (XLSX)

S1 Fig Photo-mosaic of all species portrayed with the camera installed on the crawler during the 14 months of video acquisition. Individuals in images are: (A) Eelpout (*Licenchelys* spp.). (B) Dover sole (*Microstomus pacificus*). (C) Deep sea sole (*Embassichthys bathybius*). (D) Pacific halibut (*Hippoglossus stenolepis*). (E) Rattail (*Coryphaenoides* spp.). (F) Rockfish (Sebastidae). (G) Rockfish (Sebastidae). (H) Rockfish (Sebastidae). (I) Blackfin poacher (*Bathyagonus nigripinnis*). (J) Sablefish (*Anoplopoma fimbria*). (K) Hagfish. (L) Dumbo octopus (*Grimpoteuthis* spp.). (M) Squid. (N) Buccinids (*Neptunidae*). (O) Brittle star (*Ophiuroidea*). (P) Starfish (Asteroidea). (Q) Holoturian. (R) Hermit crab. (S) Scarlet king crab. (T) Grooved tanner crab (*Chionoecetes tanneri*). (U) Male (left) and female (right) of grooved tanner crab facing each other as a part of their reproduction behaviour. (V) Male (left) carrying out a female (right) of grooved tanner crab as a part of their reproduction behaviour. (W) Small crabs, probably small individuals of grooved tanner crab. (X) Ctenophore (*Bolinopsis infundibulum*). (Y) Scyphomedusa (*Poralia rufescens*). (Z) Dinner plate jelly (*Solmissus* spp.). (AA) Traquimedusa (*Voragonema pedunculata*).
(TIF)
