# Supplementary figures and images for: Seasonal monitoring of deep-sea megabenthos in Barkley Canyon cold seep by internet operated vehicle (IOV)
Source: PLoS One. 2017 May 30;12(5):e0176917. doi: 10.1371/journal.pone.0176917 (PMC5448723; doi:10.1371/journal.pone.0176917)

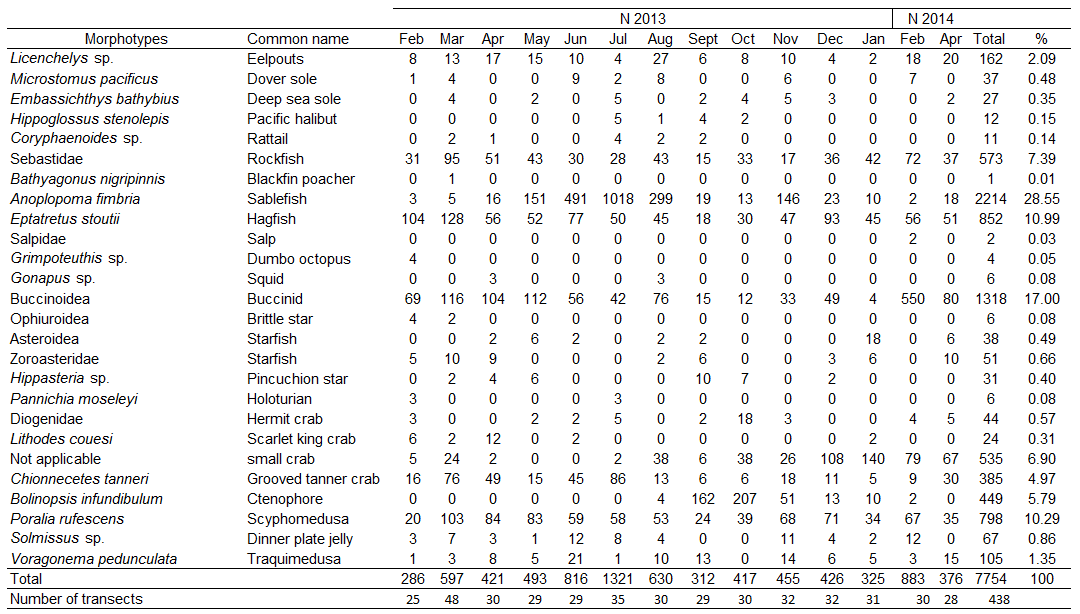

Supplement: S1 Table — (TIF) [file pone.0176917.s001.tif]

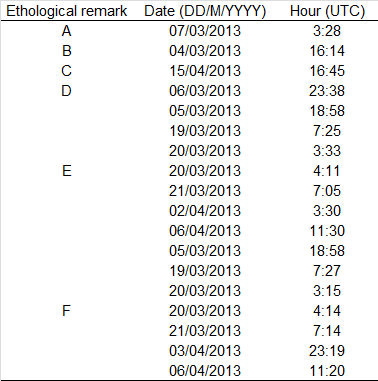

Supplement: S2 Table — (A) Rockfish (Sebatidae) agonistic display against the crawler (i.e. approaching the camera with the open mouth and then escaped). (B) Sablefish (Anoplopoma fimbria) swimming close to the crawler. (C) Male scarlet king crab (Lithodes couesi) feeding behavior and agonistic interaction with a grooved tanner crab. (D) Grooved tanner crab (Chionnecetes tanneri) agonistic display against the (i.e. an elevated body posture and chelipeds forward projection). (E) Grooved tanner crab reproduction behaviour. (F) Female grooved tanner crabs carrying eggs. (TIF) [file pone.0176917.s002.tif]

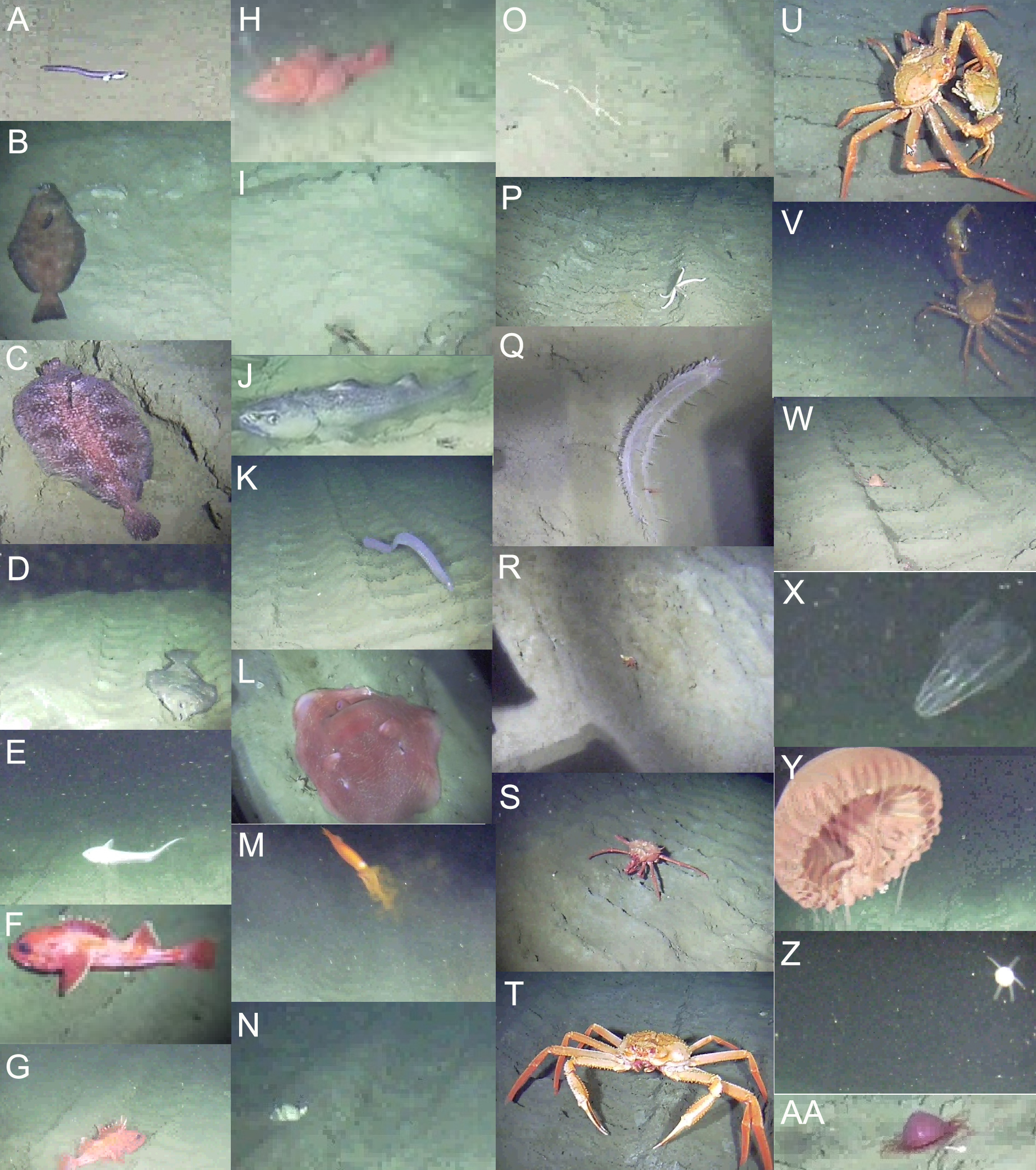

Supplement: S1 Fig — Individuals in images are: (A) Eelpout (Licenchelys spp.). (B) Dover sole (Microstomus pacificus). (C) Deep sea sole (Embassichthys bathybius). (D) Pacific halibut (Hippoglossus stenolepis). (E) Rattail (Coryphaenoides spp.). (F) Rockfish (Sebastidae). (G) Rockfish (Sebastidae). (H) Rockfish (Sebastidae). (I) Blackfin poacher (Bathyagonus nigripinnis). (J) Sablefish (Anoplopoma fimbria). (K) Hagfish. (L) Dumbo octopus (Grimpoteuthis spp.). (M) Squid. (N) Buccinids (Neptunidae). (O) Brittle star (Ophiuroidea). (P) Starfish (Asteroidea). (Q) Holoturian. (R) Hermit crab. (S) Scarlet king crab. (T) Grooved tanner crab (Chionoecetes tanneri). (U) Male (left) and female (right) of grooved tanner crab facing each other as a part of their reproduction behaviour. (V) Male (left) carrying out a female (right) of grooved tanner crab as a part of their reproduction behaviour. (W) Small crabs, probably small individuals of grooved tanner crab. (X) Ctenophore (Bolinopsis infundibulum). (Y) Scyphomedusa (Poralia rufescens). (Z) Dinner plate jelly (Solmissus spp.). (AA) Traquimedusa (Voragonema pedunculata). (TIF) [file pone.0176917.s004.tif]
